# Serial Exercise Testing and Echocardiography Findings of Patients With Kawasaki Disease

**DOI:** 10.3389/fped.2022.847343

**Published:** 2022-03-23

**Authors:** Ko-Long Lin, I-Hsiu Liou, Guan-Bo Chen, Shu-Fen Sun, Ken-Pen Weng, Chien-Hui Li, Sheng-Hui Tuan

**Affiliations:** ^1^Department of Physical Medicine and Rehabilitation, Kaohsiung Veterans General Hospital, Kaohsiung City, Taiwan; ^2^School of Medicine, Kaohsiung Medical University, Kaohsiung City, Taiwan; ^3^School of Medicine, National Yang Ming Chiao Tung University, Taipei City, Taiwan; ^4^Department of Internal Medicine, Kaohsiung Armed Forces General Hospital, Kaohsiung City, Taiwan; ^5^Congenital Structural Heart Disease Center, Department of Pediatrics, Kaohsiung Veterans General Hospital, Kaohsiung City, Taiwan; ^6^Department of Rehabilitation Medicine, Cishan Hospital, Ministry of Health and Welfare, Kaohsiung City, Taiwan; ^7^Institute of Allied Health Sciences, National Cheng Kung University, Tainan City, Taiwan

**Keywords:** Kawasaki disease, cardiopulmonary function, exercise testing, childhood, oxygen consumption, adolescence, coronary artery Z score

## Abstract

**Objective:**

Kawasaki disease (KD) is the most common form of pediatric vasculitis. We evaluated the influence of KD on cardiopulmonary function and analyzed the echocardiographic findings of patients with KD through serial follow-ups from childhood to adolescence.

**Methods:**

This was a retrospective study. We recruited patients with KD after the acute stage who underwent at least two (with >1-year interval between visits) cardiopulmonary exercise testing (CPET) and echocardiographic examinations in the last 10 years. Cardiopulmonary function was determined through CPET on a treadmill. The maximum *Z* score (Max-*Z*) of the proximal left anterior descending coronary artery or right coronary artery was determined using echocardiography. Healthy peers matched for age, sex, and body mass index with serial CPET and echocardiographic data were recruited as a control group.

**Results:**

Each group consisted of 30 participants with comparable basic characteristics. No significant differences in the variables of the first CPET were observed between the two groups. In the final CPET, the control group had a higher percentage of measured oxygen consumption (Vo_2_) at the anaerobic threshold (AT) to the predicted peak Vo_2_ (*p* = 0.016), higher percentage of measured peak Vo_2_ to the predicted peak Vo_2_ (*p* = 0.0004), and higher Vo_2_ at AT (*p* < 0.0001) than those of the KD group. No significant difference in the percentage of distribution of Max-*Z* was observed between the first and final echocardiographic examinations.

**Conclusions:**

Children with a history of KD had comparable exercise capacity to their healthy peers. However, in the follow-up, the aerobic metabolism and peak exercise load capacities of adolescents with KD were significantly lower than those of control adolescents.

## Introduction

Kawasaki disease (KD) causes inflammation of medium and small muscular arteries throughout the body and primarily affects children aged <5 years (1). KD is now the most common form of pediatric vasculitis and the leading cause of acquired coronary artery (CA) disease in children (2). After Japan and South Korea, Taiwan is the third country with the highest number of individual KD cases, with a KD incidence of approximately 28.58 to 60.08 per 100,000(3). Although most children with KD respond well to standard treatment, 5% of treated children with KD may still develop CA aneurysms (CAAs) (4), and 1% of the CAAs will eventually lead to the development of thrombosis, ischemic heart disease, myocardial infarction, or even sudden death (5).

The CA *Z* score represents how many standard deviations that a CA measurement is above or below an age-specific population mean (6). Many CA *Z* score regression equations have been proposed from large heterogeneous populations of children undergoing echocardiography to provide an objective determination of CA size abnormalities (6, 7). Most studies define a CAA to be small if the *Z* score is ≥2.5 to <5.0, large if the *Z* score is ≥5.0 to <10.0, and giant if the *Z* score is ≥10.0 (8). A previous study proved that children with KD had lower myocardial flow reserve and higher total coronary resistance than normal controls even in the absence of evidence of CA lesions (9). Recently, studies from different countries reported that a maximum CA *Z* score (Max-*Z*) of ≥2.0 in children with KD had a high predictive value for later development of CAAs (10, 11), and children with Max-*Z* ≥ 2.0 were more resistant to intravenous immunoglobulin therapy than those with normal CAs (12). Our previous study also found that children with KD with Max-*Z* ≥2.0 had significantly lower peak metabolic equivalent (MET, *p* = 0.046) and peak rate pressure product (PRPP, *p* < 0.001) than those with Max-*Z* <2.0 (13).

Most studies regarding cardiopulmonary function in patients with KD suggest that these patients have normal aerobic fitness, although fitness may decrease with the development of CAA and ischemic heart disease (14, 15). Our group and Gravel et al. both discovered that although children with KD have lower rates of myocardial perfusion while exercising, their cardiopulmonary function and exercise load capacities are similar to those of healthy children (15, 16). However, studies evaluating exercise capacity in patients with KD remain scarce and include relatively small numbers of patients. In addition, such studies include mostly children with KD. Sufficient volume of regular exercise is important to maintain health regardless of age, and the World Health Organization recommends at least an average of 60 min/day of moderate to vigorous physical activity (MVPA) across a week in children (17). Children with KD have no subjective functional limitations to physical activity in the absence of giant or multiple aneurysms (18). Nevertheless, children with KD have lower weekly MVPA and lower exercise self-efficacy evaluations compared with their healthy peers (19), which might negatively affect their cardiopulmonary function in adolescence. One recent study proved that adolescents with a KD history had significantly lower aerobic metabolism and peak exercise load capacities than controls (20). To our knowledge, no recent study has evaluated the cardiopulmonary function and CA *Z* score with a longitudinal follow-up. In this study, we aimed to observe the change in physiologic responses to exercise testing and CA *Z* score in patients with KD from childhood to adolescence through a serial follow-up. In addition, we aimed to investigate the difference of cardiopulmonary function between patients with KD and healthy controls during serial visits.

## Methods

### Participant Characteristics

This was a retrospective study, and data were obtained from a single medical center in southern Taiwan. We recruited all children with diagnosis of KD aged 10 to 18 years referred from the pediatric cardiology outpatient clinic to the department of rehabilitation from May 2011 to May 2021 for regular follow-up with cardiopulmonary exercise testing (CPET). The following additional inclusion criteria were applied: (a) underwent at least two symptom-limited treadmill exercise tests, separated by >1 year; (b) underwent a complete transthoracic echocardiographic examination; and (c) underwent a standard 12-lead electrocardiography. Patients with KD with (a) significant structural heart disease, (b) moderate to severe cardiac valvular disease, (c) significant arrhythmia, (d) ventricular hypertrophy, and (e) concurrent known pulmonary disease or other diseases that might interfere with their cardiopulmonary function were excluded. Age-, sex-, and body mass index (BMI)–matched children referred to the pediatric cardiology outpatient clinic during the same period for chest pain, palpitation, or dyspnea on exertion, who (1) were diagnosed not to have any specific disease after series of examinations, including physical examinations by pediatricians, CPET, echocardiography, and 12-lead electrocardiography and (2) happened to receive at least two CPETs (separated by >1 year), were recruited as a control group. The study was conducted following the principles outlined in the Helsinki Declaration and was approved by the institutional review board of Kaohsiung Veterans General Hospital (no. VGHKS17-CT11-11). This study adhered to the STROBE (STrengthening Reporting of OBservational studies in Epidemiology) reporting guidelines.

### Treadmill Exercise Testing

We used symptom-limited exercise testing, which was composed of a treadmill, a flow module, a gas analyzer, and an electrocardiographic monitor (Metamax 3B; Cortex Biophysik GmbH Co., Leipzig, Germany), to measure the participants' exercise capacity. The entire test was supervised by an experienced physiatrist with more than 15 years of experience in CPET (K.-L.L.). Before the treadmill exercise test, each participant was familiarized to the procedures and testing equipment through a demonstration. The purpose of the testing was explained to the participants and their families before obtaining their informed consent (verbal consent from participants and written consent from family members). All participants underwent exercise testing according to the Bruce ramp protocol, as suggested by the American College of Sports Medicine (ACSM). We stopped the test when the participants demonstrated subjective unbearable symptoms, when they could no longer continue, or when they attained maximal effort according to the ACSM definition (21). The oxygen consumption (Vo_2_) and carbon dioxide production (Vco_2_) during the testing were measured using the breath-by-breath method. In addition, blood pressure (BP), heart rate (HR), HR reserve (defined as peak HR minus baseline HR), and respiratory exchange ratio (RER) were measured throughout the exercise test. Oxygen pulse was calculated as the oxygen uptake divided by HR (as an indicator of the stroke volume and oxygen extraction by cells) (22), and PRPP was calculated as the peak systolic BP multiplied by the peak HR (as an indicator of the myocardial oxygen demand and myocardial workload during exercise) (23). The Vo_2_ at the anaerobic threshold (AT Vo_2_) and the maximum oxygen uptake measured at peak exercise (peak Vo_2_) were also determined. AT Vo_2_% and peak Vo_2_% were defined as the percentage of the measured Vo_2_ at the AT to the predicted peak Vo_2_ and the percentage of the measured peak Vo_2_ to the predicted peak Vo_2_, respectively, after comparing with the normal standards for cardiopulmonary responses to exercise in Taiwan (24). The measured Vo_2_ was divided by a constant (3.5 mL/kg per minute) to derive the MET. The AT was determined using the VE/Vo_2_ and VE/Vco_2_ methods (25).

### Pulmonary Function Test

We used spirometry at rest to measure the pulmonary function of the participants. The forced vital capacity (FVC), forced expiratory volume in 1 s (FEV1), and maximal voluntary ventilation (MVV) were measured. We divided the measured FVC by the predicted FVC (FVCP), the measured FEV1 by the predicted FEV1 (FEV1P), and the measured MVV by the predicted MVV (MVVP). The predicted value of each spirometric parameter was calculated using the spirometric reference equations for healthy children and adolescents in Taiwan (26).

### Echocardiography and CA Z Score

Only the participants with KD received the echocardiography, and all the echocardiography of each participant with KD was done within 1 month before or after the date of CPET. Two experienced pediatric cardiologists used a >5-MHz sector probe for echocardiography based on the standard measurement methods for pediatric CA as recommended by the Japanese Society of Kawasaki Disease. All patients with KD were examined in the supine or right decubitus position and underwent complete two-dimensional echocardiographic studies with color flow and spectral Doppler examination. The intraluminal diameters of the CA segments were measured from inner edge to inner edge. The right CA (RCA) and left anterior descending CA (LCA) were measured 3 to 5 mm distal to their origins in the parasternal short-axis view (27). We also measured routinely examined cardiac structures in patients with KD, including the valves, left ventricular (LV) diameter, aortic root (AO) diameter, LV diameter at end diastole (LVIDd), and LV diameter at end systole (LVIDs), according to the guidelines and standards for performing pediatric echocardiography of the American Society of Echocardiography (28).

To calculate the CA *Z* score, we used the equation derived using the lambda–mu–sigma method, as proposed by Kobayashi et al. The equation was established after collecting data from 3,851 healthy Japanese children aged from 0 month to 18.9 years, and it can be used to estimate the sex-specific *Z* score of each internal CA diameter with a goodness of fit comparable to that of previously reported regression models (8). A Microsoft Excel–based *Z* score calculator (ZSP version 4) is freely available online (http://raise.umin.jp/zsp/calculator/). The CA *Z* score was computed using the ZSP version 4 calculator after entering sex-specific data on age, body height, body weight, body surface area calculated using the Haycock formula, and CA diameter measured on echocardiography. The highest CA *Z* score of the proximal LCA or RCA was recorded as the Max-*Z*.

### Statistical Analysis

We used Statistical Package for the Social Sciences for Windows, version 19.0 (IBM Corp., Armonk, NY, USA) for all analyses. We did not determine the minimum sample size, given that this was a retrospective study by using data from the database, and we took all cases met the recruited criteria in the targeted years as many as possible. Continuous data were expressed as mean ± standard deviation, and categorical variables were presented as absolute numbers or percentages. Normality and homoscedasticity were examined before each analysis. We used the independent *t-*test to compare the basic characteristics and outcomes between the patients with KD and the control at the initial and the final visits. For the differences between initial and final values for each exercise test and echocardiographic variable of patients with KD, we used the paired Student *t-*test to compare the data. *p* < 0.05 was used to denote statistical significance.

## Results

**Figure 1:**
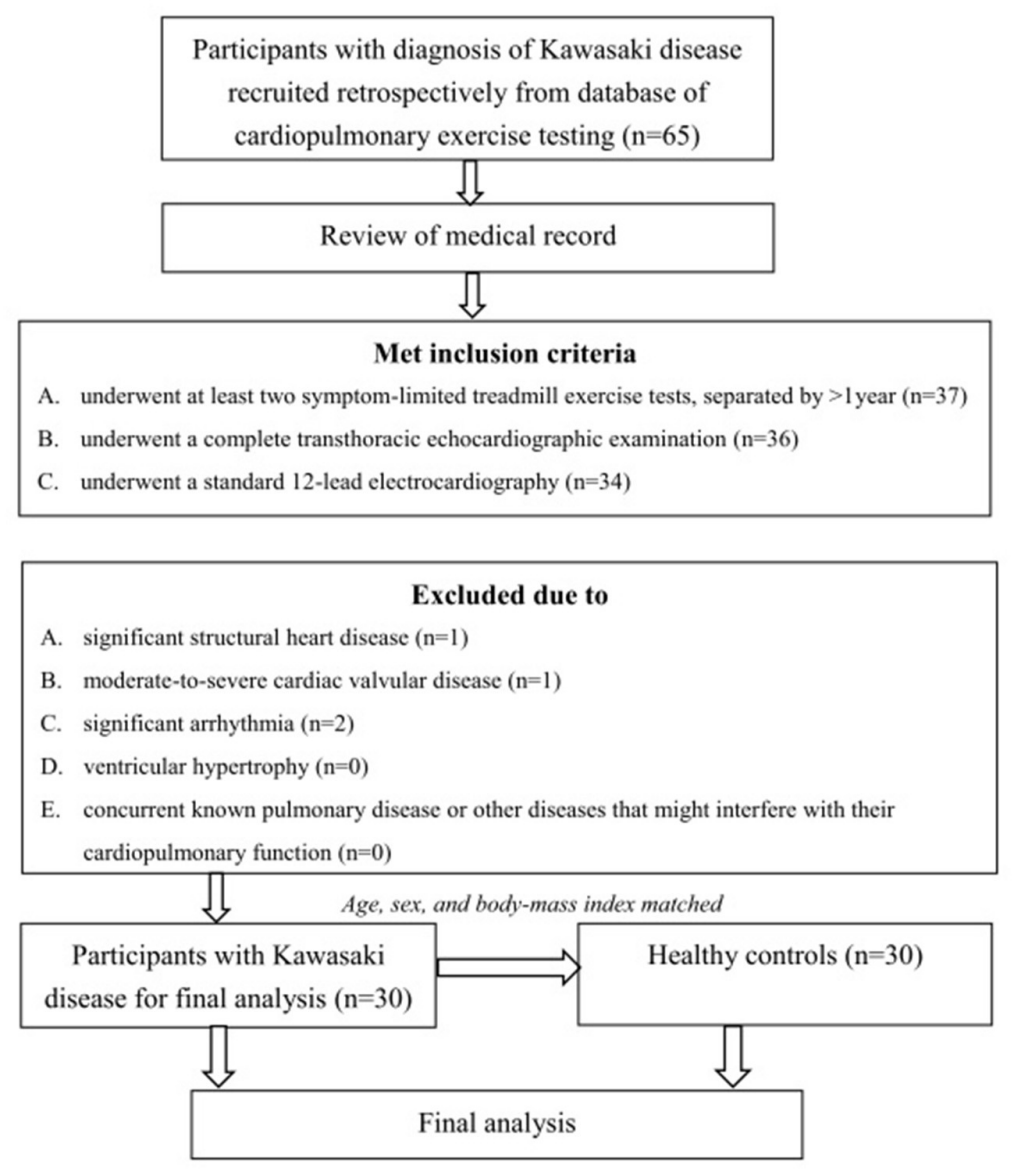
Algorithm of the study. A total of 34 patients met the inclusion criteria. Among them, one patient had moderate valvular disease, one patient had significant cardiac structural problems, and two patients had significant arrhythmia. Therefore, 30 participants with KD were recruited for the final analysis. Thirty age-, sex-, and BMI-matched healthy participants were recruited as the control group.

A total of 34 patients met the inclusion criteria. Among them, one patient had moderate valvular disease, one patient had significant cardiac structural problems, and two patients had significant arrhythmia. Therefore, 30 participants with KD were recruited for the final analysis. In addition, 30 age-, sex-, and BMI-matched healthy participants were recruited as the control group (Figure 1).

### Demographic Characteristics

Table 1 shows the demographic characteristics of the KD and control groups. The mean ages of patients with KD and controls were 12.68 ± 3.77 and 13.06 ± 3.64 years at the time of the first CPET and 16.71 ± 4.83 and 16.12 ± 2.70 years at the time of the final CPET, respectively. The average intervals between the first and last CPET were 4.03 ± 2.82 years in the KD group and 3.06 ± 2.24 years in the control group (*p* = 0.146). No significant differences in sex, age, weight, height, BMI, body fat, systolic and diastolic BPs, resting HR, and all routinely examined spirometric parameters (FVC, FVCP, FEV1, FEV1P, MVV, and MVVP) were found between the KD and control groups.

**Table 1:** Demographic characteristics of patients with Kawasaki disease and control.

	**KD group 1st CPET** **(*n* = 30)**	**Control group 1st CEPT** **(*n* = 30)**	***p-*value^**a**^**	**KD group last CPET** **(*n* = 30)**	**Control group last CEPT** **(*n* = 30)**	***p-*value^**a**^**
Gender (M:F)	19:11	19:11	1.000	19:11	19:11	1.000
Age (years)	12.68 ± 3.77 (11.32–14.03)	13.06 ± 3.64 (11.88–14.31)	0.693	16.71 ± 4.83 (14.94–17.58)	16.12 ± 2.70 (14.48–16.95)	0.562
Height (cm)	149.11 ± 15.28 (144.05–156.68)	151.52 ± 17.55 (151.98–162.66)	0.548	159.93 ± 15.50 (154.54–166.34)	161.10 ± 14.23 (162.02–169.93)	0.780
Weight (kg)	45.38 ± 12.95 (41.49–52.10)	49.15 ± 16.02 (47.39–57.23)	0.290	54.56 ± 17.32 (49.66–62.72)	57.36 ± 16.36 (54.09–67.29)	0.554
BMI (kg/m^2^)	19.75 ± 3.08 (18.78–21.18)	20.82 ± 9.35 (19.53–22.52)	0.246	20.66 ± 4.80 (19.38–22.83)	21.37 ± 4.76 (19.17–23.72)	0.595
Body fat (%)	17.62 ± 7.76 (14.98–21.50)	20.79 ± 9.35 (14.67–23.89)	0.145	18.76 ± 8.75 (16.76–23.04)	22.81 ± 8.15 (18.26–26.09)	0.092
Resting SBP (mm Hg)	111.50 ± 11.94 (108.16–117.19)	111.66 ± 15.89 (105.42–120.30)	0.964	118.00 ± 15.07 (114.61–125.84)	117.46 ± 16.21 (113.97–125.94)	0.901
Resting DBP (mm Hg)	65.15 ± 8.34 (62.66–68.69)	64.66 ± 8.98 (58.82–67.75)	0.819	67.50 ± 8.40 (66.40–70.96)	67.29 ± 8.26 (64.56–71.63)	0.929
Resting HR (bpm)	84.59 ± 14.12 (78.60–89.73)	88.68 ± 15.39 (79.85–91.29)	0.258	80.50 ± 12.91 (75.10–84.64)	84.58 ± 14.39 (75.59–89.93)	0.286
FVC (L)	2.68 ± 1.11 (2.24–3.21)	2.42 ± 1.26 (2.18–3.35)	0.402	3.34 ± 0.85 (2.98–3.71)	3.22 ± 1.17 (1.90–3.42)	0.708
FVCP (%)	104.99 ± 28.91 (85.04–112.97)	97.68 ± 21.33 (71.50–102.89)	0.298	101.05 ± 16.21 (90.82–101.19)	98.19 ± 21.92 (67.80–100.62)	0.634
FEV1 (L)	2.42 ± 1.00 (1.93–2.81)	2.19 ± 1.17 (1.89–3.00)	0.417	2.91 ± 0.86 (2.62–3.29)	2.61 ± 1.41 (1.68–3.11)	0.390
FEV1P (%)	110.62 ± 34.16 (83.25–117.74)	100.95 ± 24.84 (72.71–106.37)	0.243	100.54 ± 19.04 (91.55–104.41)	99.56 ± 30.08 (75.73–99.83)	0.899
FEV1/FVC (%)	91.26 ± 10.42 (82.74–92.48)	90.87 ± 7.28 (75.05–93.78)	0.869	86.45 ± 8.72 (84.79–92.43)	89.39 ± 8.51 (80.06–96.11)	0.294
MVV (L)	62.29 ± 28.80 (49.01–73.33)	65.31 ± 33.58 (44.98–82.27)	0.718	79.57 ± 30.66 (69.26–94.57)	76.60 ± 35.53 (42.86–80.32)	0.786
MVVP (%)	106.30 ± 54.80 (67.14–114.30)	129.39 ± 57.54 (78.18–141.90)	0.143	81.52 ± 38.07 (63.53–84.16)	101.39 ± 66.34 (62.76–102.55)	0.311

*Data are the mean ± standard deviation*.

*M, male; F, female; KD, Kawasaki disease; BMI, body mass index; SBP, systolic blood pressure; DBP, diastolic blood pressure; HR, heart rate; FVC, functional vital capacity; FVCP, percentage of predicted forced vital capacity; FEV1, force expiratory volume at 1 minute; FEV1P, percentage of predicted forced expiratory volume at 1 minute; MVV, maximal voluntary ventilation; MVVP, percentage of predicted maximal voluntary ventilation*.

*The subscript ‘a' indicates to the p value of independent-t test between KD group and control group*.

### Performance in Treadmill Exercise Testing

Intragroup and intergroup comparisons of the initial and final exercise testing data in the KD and control groups are presented in Table 2.

**Table 2:** Intragroup and intergroup comparisons between the initial and the final exercise testing data in patients with Kawasaki disease and control group.

**Variables**		**First CPET**	**Last CPET**	***P*-value^**†**^**	**Change per year**
Peak Vo_2_ (mL/min)	KD	1,566.43 ± 469.05 (1,383.26–1,741.98)	1,880.57 ± 578.62 (1,663.45–2,100.87)	<0.001^*^	112.44 ± 216.98 (32.85–192.03)
	Control	1,670.94 ± 457.37 (1,462.75–1,879.14)	1,928.72 ± 456.38 (1,720.98–2,136.46)	0.075	78.13 ± 167.38 (1.94–154.32)
	*p-*value^‡^	0.425	0.764		0.544
Peak MET	KD	9.57 ± 1.79 (9.07–10.38)	9.64 ± 1.87 (9.04–10.43)	0.761	0.03 ± 0.59 (−0.19 to 0.24)
	Control	9.12 ± 1.69 (8.35–9.89)	9.26 ± 2.05 (8.33–10.20)	0.813	0.05 ± 0.87 (−0.35 to 0.44)
	*p*-value^‡^	0.230	0.400		0.915
Peak Vo_2_(% predicted)	KD	93.41 ± 25.43 (83.51–100.03)	76.17 ± 14.45 (70.82–81.44)	<0.001^*^	−5.75 ± 10.77 (−9.70 to −1.80)
	Control	99.61 ± 24.77 (89.24–110.12)	92.23 ± 18.36 (85.21–102.56)	0.195	−1.82 ± 8.66 (−3.42 to 2.68)
	*p*-value^‡^	0.343	0.0004^*^		0.1248
Vo_2_ at AT(mL/min)	KD	1,091.07 ± 299.86 (965.06–1,193.70)	1,047.60 ± 316.61 (912.54–1,149.94)	0.257	−14.61 ± 92.29 (−48.47 to 19.24)
	Control	1,136.07 ± 276.84 (1,010.05–1,262.09)	1,388.29 ± 349.05 (1,229.40–1,547.18)	0.013^*^	92.95 ± 163.85 (18.36–167.53)
	*p*-value^‡^	0.504	<0.0001^*^		0.011^*^
MET at AT	KD	6.69 ± 1.15 (6.31–7.19)	6.38 ± 1.26 (5.93–6.89)	0.208	−0.09 ± 0.72 (−0.36 to 0.17)
	Control	6.21 ± 0.83 (5.84–6.59)	6.65 ± 1.43 (6.00–7.30)	0.238	0.22 ± 0.81 (−0.14 to 0.59)
	*p*-value^‡^	0.080	0.539		0.147
AT Vo_2_ (% predicted)	KD	51.98 ± 11.19 (47.87–56.08)	43.54 ± 17.13 (37.26–49.83)	0.027^*^	−3.19 ± 5.98 (−5.39 to −1.00)
	Control	52.61 ± 10.39 (43.88–53.34)	53.24 ± 12.69 (46.46–58.01)	0.588	0.24 ± 6.03 (−0.78 to 4.72)
	*p*-value^‡^	0.822	0.016^*^		0.03^*^
Peak RER	KD	1.15 ± 0.08 (1.12–1.18)	1.18 ± 0.11 (1.15–1.23)	0.123	0.01 ± 0.05 (−0.01 to 0.03)
	Control	1.21 ± 0.14 (1.14–1.27)	1.18 ± 0.12 (1.13–1.24)	0.517	−0.01 ± 0.07 (−0.05 to 0.02)
	*p*-value^‡^	0.107	0.778		0.180
Peak HR (bpm)	KD	176.71 ± 16.44 (170.58–183.16)	175.94 ± 8.79 (173.39–179.84)	0.788	1.16 ± 12.93 (−3.57 to 5.91)
	Control	177.14 ± 11.74 (171.80–182.48)	178.14 ± 8.15 (174.43–181.85)	0.750	0.38 ± 3.79 (−1.35 to 2.10)
	*p*-value^‡^	0.950	0.529		0.788
HRR (bpm)	KD	29.44 ± 9.20 (26.14–34.50)	30.36 ± 11.01 (24.80–32.80)	0.619	−0.45 ± 6.38 (−3.28 to 2.38)
	Control	28.25 ± 11.82 (23.01–36.99)	28.45 ± 5.59 (26.12–33.56)	0.150	0.76 ± 7.93 (−2.96–4.47)
	*p*-value^‡^	0.681	0.400		0.588
PRPP	KD	28,704.74 ± 5,330.16 (27,019.45–30,964.55)	28,870.62 ± 5,011.89 (27,066.38–30,881.30)	0.842	2.06 ± 2,547.08 (−932.21–936.34)
	Control	27,454.86 ± 5,741.98 (24,841.14–30,068.57)	29,026.90 ± 6,131.19 (26,236.02–31,817.79)	0.396	338.74 ± 4,743.14 (−1,820.30–2,497.80)
	*p*-value^‡^	0.330	0.973		0.768
Peak O_2_ pulse (mL/beat)	KD	8.85 ± 2.55 (7.85–9.79)	10.71 ± 3.35 (9.41–11.93)	<0.001^*^	0.60 ± 1.29 (0.13–1.07)
	Control	9.38 ± 2.36 (8.31–10.46)	10.81 ± 2.45 (9.70–11.93)	0.061	0.43 ± 0.85 (0.04–0.82)
	*p*-value^‡^	0.434	0.874		0.601

*Data are the (upper row) mean ± standard deviation and (lower row) 95% confidence interval*.

*KD, Kawasaki disease; CPET, cardiopulmonary exercise testing; peak Vo_2_, peak oxygen consumption; MET, metabolic equivalent; peak MET, largest MET during whole exercise testing; % of peak predicted, percentage of predicted peak Vo_2_; AT MET, MET at the point of anaerobic threshold; HR, heart rate; peak RER, largest respiratory exchange ratio during whole exercise testing; HRR, heart rate reserve; PRPP, peak rate-pressure product*.

* *p < 0.05*.

† *Refers to the p-value of paired t-test between the first and the last CPET of the KD or the control group*.

‡ *Refers to the p-value of independent t-test between the KD group and the control group*.

### Serial CPET Data of the KD Group

The average number of exercise tests in the KD group was 2.8 per patient. The peak Vo_2_ and oxygen pulse significantly increased (both *p* <0.001), whereas the peak MET remained the same from the first to the final CPET (*p* = 0.761). Both peak Vo_2_% (from 93.41% ± 25.43% to 76.17% ± 14.45%, *p* <0.001) and AT Vo_2_% (from 51.98% ± 11.19% to 43.54% ± 17.13%, *p* = 0.027) significantly declined from first to the final CPET (95% confidence interval of change of peak Vo_2_% and AT Vo_2_% yearly was −9.70 to −1.80 and −5.39 to −1.00, respectively). No significant changes were observed in any of the other exercise variables, including Vo_2_ at AT, MET at AT, peak RER, peak HR, and PRPP, between the first and final CPET.

### Serial CPET Data of the Control Group

The average number of exercise tests in the control group was 2.2 per participant. No significant changes were observed in all studied exercise variables between the first and final CPET, except for Vo_2_ at AT, which significantly increased from 1,136.07 ± 276.84 to 1,388.29 ± 349.05 mL/min (*p* = 0.013).

### Intergroup Comparisons Between the KD and Control Groups

The KD and control groups presented no significant differences in the variables of the first CPET. However, in the final CPET, the control group had higher peak Vo_2_% (*p* = 0.0004), AT Vo_2_% (*p* = 0.016), and Vo_2_ at AT (*p* < 0.0001) than the KD group. With regard to the change in CPET variables per year, the Vo_2_ at AT and AT Vo_2_% decreased in the KD group but increased in the control group (*p* = 0.011 and 0.03, respectively).

### Echocardiographic Findings

Table 3 shows the echocardiographic findings of all recruited patients with KD at the first and last visits. The proximal RCA *Z* score, proximal LCA *Z* score, and Max-*Z* were 0.07 ± 1.07 (median = 0.11, range = −1.93 to 2.49), 0.33 ± 1.01 (median = −0.24, range = −1.77 to 2.90), and 1.11 ± 0.72 (median = 0.96, range = 0.18 to 2.90) in the first examination and 0.01 ± 1.26 (median = 0.06, range = −1.98 to 2.83), 0.20 ± 1.08 (median = −0.49, range = −1.50 to 2.89), and 1.23 ± 0.70 (median = 1.04, range = 0.40 to 2.89) in the last examination, respectively. In the first examination, two patients with KD had Max-*Z* > 2.5, and one patient had a Max-*Z* of 2.0–2.5. In the last examination, four patients with KD had Max-*Z* > 2.5, and one patient had a Max-*Z* of between 2.0 and 2.5. No significant difference in the percentage of distribution of Max-*Z* was noted between the first and final CPET (*p* = 0.689, χ^2^ test). Both LVIDd (*p* = 0.013) and LVIDs (*p* = 0.039) significantly increased from the first to the last examination, whereas the other echocardiographic variables measured during routine examinations, including LV shortening fraction, LA diameter, and AO diameter, showed no significant differences.

**Table 3:** Corresponding echocardiogrpahic findings of the first and the last cardiopulmonary exercise testing in participants with Kawasaki disease.

	**First examination (*n* = 30)**	**Last examination (*n* = 30)**	**Mean difference from last data minus first data**	***p*-value**
LVIDd (cm)	3.93 ± 0.57 (3.71–4.13)	4.27 ± 0.54 (4.04–4.45)	0.34 ± 0.69 (0.08–0.61)	0.013*
LVIDs (cm)	2.35 ± 0.36 (2.23–2.49)	2.51 ± 0.36 (2.36–2.63)	0.16 ± 0.40 (0.01–0.32)	0.039*
LV shortening FR (%)	39.91 ± 7.00 (36.81–42.21)	40.98 ± 6.98 (38.48–43.61)	1.07 ± 9.74 (−2.64–4.78)	0.559
LA (cm)	2.01 ± 0.40 (1.86–2.15)	2.20 ± 0.47 (2.03–2.37)	0.19 ± 0.51 (−0.01–0.38)	0.056
AO (cm)	1.81 ± 0.32 (1.68–1.92)	1.86 ± 0.40 (1.71–2.01)	0.06 ± 0.41 (−0.10–0.21)	0.478
RCA diameter (cm)	0.28 ± 0.05 (0.26–0.30)	0.29 ± 0.05 (0.28–0.31)	0.15 ± 0.67 (−0.10–0.41)	0.229
LCA diameter (cm)	0.29 ± 0.05 (0.27–0.31)	0.32 ± 0.05 (0.30–0.34)	0.24 ± 0.50 (0.05–0.43)	0.014*
RCA *Z* score by ZSP	0.78 ± 0.68 (0.52–1.04)	0.93 ± 0.81 (0.63–1.23)	−0.70 ± 1.29 (−0.56–0.42)	0.775
LCA *Z* score by ZSP	0.76 ± 0.74 (0.48–1.04)	0.90 ± 0.81 (0.68–1.12)	0.13 ± 1.11 (−0.29–0.55)	0.536
Max-*Z* by ZSP	1.03 ± 0.19 (0.96–1.11)	1.21 ± 0.70 (0.95–1.47)	0.13 ± 0.88 (−0.21–0.46)	0.442

*Data are the mean ± standard deviation (95% confidence interval). LVIDd, diastolic left ventricular (LV) internal diameter; LVIDs, systolic left ventricular internal diameter; LV shortening FR, LV shortening fraction = (LVIDd-LVIDs)/ LVIDd; LA, diameter of left atrium; AO, diameter of aortic root; RCA, right coronary artery measured 3 to 5 mm distal to its origins in the parasternal short-axis view; LCA, left anterior descending coronary artery measured 3 to 5 mm distal to its origins in the parasternal short-axis view; RCA/LCA Z score by ZSP, coronary Z score of RCA/LCA calculated by ZSP version 4 calculator; Max-Z by ZSP, maximum Z score of the LCA or RCA by ZSP version 4 calculator*.

* *p < 0.05*.

## Discussion

This is the first study to investigate the cardiopulmonary function and aerobic fitness of patients with KD through serial CPET and echocardiographic evaluations. We observed that patients with KD had lower peak Vo_2_%, AT Vo_2_%, and Vo_2_ at AT in the final CPET than the control group. We also found that peak Vo_2_% and AT Vo_2_% in the KD group significantly decreased between two CPETs in the study period compared with those in the control group.

Similar to previous findings of ours (16) and other teams (29, 30), we discovered that the aerobic metabolism and peak exercise load capacities of patients with KD were not significantly different from those of the control group in the first CPET. Such findings could be discussed by two explanations. One is that patients with KD might resolve with no sequelae in a long-term follow-up (4), and the other is that the patients recruited in those studies were those with lower cardiopulmonary risk. Given that none of these studies provided the information about the CA involvement at the acute stage of KD, we could only make the above assumption. However, this conclusion was not applicable in the last CPET in our study. In the present study, the average ages of the KD group in the first and last CPET were 12.68 ± 3.77 and 16.71 ± 4.83 years, respectively. By using the definition by the Centers for Disease Control and Prevention of the United States (31), 28 of 30 (93.3%) and 2 of 30 (6.7%) KD participants were young teens (12–14 years of age) and teenagers (15–17 years of age), respectively, during the first CEPT in this current study. In our previous study, the average age of patients with KD was 12.27 ± 3.76 years. In other words, most of the participants in our previous study and in the first CPET of the present study were young teens, whereas the participants in the final CPET of the present study were all teenagers who had been diagnosed longer with KD. Meanwhile, we did not observe significant changes in Max-*Z*, and the percentage of participants with Max-*Z* > 2.5 was not significantly different between the first and final echocardiography in patients with KD. None of the patients with KD had CAA in both the first and final echocardiographic examinations. One assumption is that this discrepancy in the CPET findings between childhood and adolescence might be due to the different responses to physical activity at different developmental stages. The aerobic capacity of children and consequently their exercise tolerance increase as they grow, especially during puberty when a growth spurt occurs (32). The absolute maximal aerobic power (in liters per minute) fairly linearly increases in boys until approximately age 16 years, whereas it increases in girls until approximately age 13 years and plateaus during adolescence (32, 33). Aerobic capacity in youth increases with activity of sufficient intensity (particularly MVPA), and the maximal stroke volume, blood volume, and levels of oxidative enzymes improve after exercise training (34). Studies regarding the physical activity levels of youth after KD are scarce and controversial (19, 20, 35). One study reported that children with KD had lower weekly MVPA and lower exercise self-efficacy evaluations than their healthy peers (19). Owing to the growth spurt, the physiological adaptation to MVPA might be greater in adolescence (36). Therefore, the difference in exercise tolerance and aerobic fitness from healthy peers was observed only in adolescents rather than in children with KD. Future studies are warranted to determine whether the daily activity levels of youth who once had KD differ from those of their healthy peers and to investigate the effects of previous KD on cardiopulmonary function.

Another assumption regarding the discrepancy in the CPET findings between childhood and adolescence is that it might be related to the long-term effects of KD on the circulatory system. After the acute stage, there might still be ongoing intense inflammatory process, resulting in myointimal proliferation and organizing thrombus in the CAs with recanalization (37). Studies also found pathologic changes in the myocardium as a result of injury during the acute inflammatory phase of KD (38, 39). Although we did not observe any CAA and the percentage of distribution of Max-*Z* > 2.0 showed no significant difference in the serial echocardiographic follow-up, it did not mean that the vascular structure of the participants with KD remained the same from childhood to adolescence. Studies that performed ultrasound cardiography and selective coronary angiography (CAG) have proven that an abnormal vascular structure is present at the previous site of an acute CAA because of healing-related intimal thickening, although small CAAs in the acute phase of KD revert to a normal appearance in the convalescent phase (40, 41). Iemura et al. discovered that the CAs of patients with KD still had thickened inner walls, significant contractions, and poor diastolic function 10 years after KD treatment (42). One recent study that performed optical coherence tomography showed that intimal thickening and disruption of the media occur in CAs with normal CAG findings in the acute and convalescent phases of KD (43). In several reports, patients with KD with angiographically normal CA later developed cardiovascular disorders after adolescence (44, 45). These studies support the findings of our study that the effects of KD on the cardiovascular system remain years later, which might influence the cardiopulmonary function of patients with KD who are entering adolescence.

Although our study results indicated that adolescents with KD had lower aerobic capacity and peak exercise tolerance, patients with KD should still engage in normal exercise activities. Our findings might have resulted from the relative lack of MVPA of participants with KD in their adolescence, as discussed previously. The World Health Organization recommends at least an average of 60 min/day of MVPA, mostly aerobic activity, across the week in children and adolescents aged 5 to 17 years (46). The ACSM defines vigorous physical activity as >6 MET. The average peak METs in the KD group in our study were 9.12 ± 1.69 and 9.26 ± 2.05 in the first and final CPET, respectively, exceeding the requirements for most vigorous exercises. This indicates that the KD group in our study can engage in normal vigorous daily activities. According to the guidelines of the American Heart Association and the Japanese Ministry of Health, patients with KD should have no limitations after the acute stage unless they are classified into the high-risk group (with persistent, stenotic CAA with or without ischemia) (2, 47). Therefore, it is crucial to emphasize the advantages of a physically active lifestyle and encourage active and regular exercise after the acute stage of KD.

This study must be viewed in light of some limitations. First, retrospective nature of the study makes it open to bias, and the timing of follow-up varied. Ideally, participants with KD should receive follow-up, including outpatient clinic visit, echocardiography, and CPET annually, but some of them did not have good compliance to the follow-up in the real-world setting. Moreover, the controls were healthy peers who happened to receive at least two CPETs from our database. Therefore, KD participants and controls were not seen at the same interval. Besides, although all recruited participants with KD underwent a complete transthoracic echocardiographic examination within 1 month before or after the date of CPET, there still might be uncontrolled bias. Second, we recruited the control group through an age-, sex-, and BMI-matching method. Although no significant statistical difference in basic characteristics was noted, differences in body composition might have existed between the KD and control groups. Third, most of the patients with KD had normal Max-*Z*, and none of them presented CAA in the serial examinations. As no patients with KD underwent exercise testing in the acute stage, we could not confirm whether the normal Max-*Z* in the KD group was due to regression of CA dilatation after the acute stage or other reasons such as discrepancies between CA growth and somatic growth. Moreover, we did not have data about the initial CA involvement in the acute stage of KD. Therefore, we could not stratify the population following the severity of CA involvement, which relates to time-dependent occurrence of cardiac event (48) and might interfere with the cardiopulmonary function in the later life. Fourth, we recruited participants from a single medical center in southern Taiwan. A larger cross-national study is needed for further evaluation. Lastly, we used ZSP version 4, a calculator based on data from multiple centers across Japan, to calculate the CA *Z* score as there is no well-accepted equation for calculating the CA *Z* score based on Taiwanese children aged >6 years. The Max-*Z* calculated by ZSP version 4 might not fully reflect the true CA condition of Chinese children with KD.

## Conclusion

In this study, we found that children with a KD history had comparable exercise capacity to their healthy peers. However, in the follow-up, the aerobic metabolism and peak exercise load capacities of adolescents with KD were significantly lower than those of control adolescents. This discrepancy in the CPET findings between childhood and adolescence might be due to the different responses to physical activity at different developmental stages and the long-term effects of KD on the circulatory system. Further studies evaluating the direct effect of physical activity and CA characteristics on the exercise capacity of patients with KD are warranted. In addition, children and adolescents with KD without CAAs can safely engage in MVPA because their peak exercise capacity is much higher than the recommended vigorous-intensity physical activity levels as we observed in this study.

## Data Availability Statement

The raw data supporting the conclusions of this article will be made available by the authors, without undue reservation.

## Ethics Statement

The study was carried out by following the principles outlined in the Helsinki Declaration and was approved by the Institutional Review Board of Kaohsiung Veterans General Hospital (number: VGHKS17-CT11-11). Written informed consent to participate in this study was provided by the participants' legal guardian/next of kin.

## Author Contributions

K-LL, I-HL, and S-HT: conceptualization. K-LL, C-HL, and S-HT: data curation. G-BC, S-FS, and C-HL: methodology. K-LL and I-HL: resources. S-FS and K-PW: supervision. K-LL and G-BC: writing–original draft. I-HL and S-HT: writing–review and editing. All authors contributed to the article and approved the submitted version.

## Funding

This study was supported by the research funds of Kaohsiung Veterans General Hospital, Number: KSVGH-IGA-110-03.

## Conflict of Interest

The authors declare that the research was conducted in the absence of any commercial or financial relationships that could be construed as a potential conflict of interest.

## Publisher's Note

All claims expressed in this article are solely those of the authors and do not necessarily represent those of their affiliated organizations, or those of the publisher, the editors and the reviewers. Any product that may be evaluated in this article, or claim that may be made by its manufacturer, is not guaranteed or endorsed by the publisher.

## Abbreviations

KD: Kawasaki disease
CA: coronary artery
CAA: coronary artery aneurysm
Max-*Z*: maximum coronary artery *Z* score
CPET: cardiopulmonary exercise testing
ACSM: American College of Sports Medicine
Vo_2_: oxygen consumption
Vco_2_: carbon dioxide production
BP: blood pressure
HRR: heart rate reserve
RER: respiratory exchange ratio
PRPP: peak rate pressure product
AT: anaerobic threshold
AT Vo_2_%: percentage of the measured Vo_2_ at anaerobic threshold to the predicted peak Vo_2_
peak Vo_2_%: percentage of measured peak Vo_2_ to the predicted peak Vo_2_
MET: metabolic equivalent
FVC: forced vital capacity
FVCP: measured FVC divided by the predicted FVC
FEV1: forced expiratory volume in 1 second
FEV1P: measured FEV1 divided by the predicted FEV1
MVV: maximal voluntary ventilation
MVVP: measured MVV divided by the predicted MVV
RCA: right coronary artery
LCA: left anterior descending coronary artery
LVIDd/LVIDs: LV diameter at end diastole/systole.
